# Assessment of Transformed Properties *In Vitro* and of Tumorigenicity *In Vivo* in Primary Keratinocytes Cultured for Epidermal Sheet Transplantation

**DOI:** 10.1155/2011/936546

**Published:** 2010-09-19

**Authors:** A. Thépot, A. Desanlis, E. Venet, L. Thivillier, V. Justin, A. P. Morel, F. DeFraipont, M. Till, V. Krutovskikh, M. Tommasino, O. Damour, P. Hainaut

**Affiliations:** ^1^Banque de Tissus et Cellules, Hôpital E. Herriot, 69437 Lyon cedex 03, France; ^2^Centre International de Recherche sur le Cancer, 69372 Lyon cedex 08, France; ^3^Centre Léon Bérard, 69373 Lyon cedex 03, France; ^4^Centre Hospitalier Universitaire, 38043 Grenoble cedex 09, France; ^5^Laboratoire de Cytogénétique HFME, 69677 Bron, France

## Abstract

Epidermal keratinocytes are used as a cell source for autologous and allogenic cell transplant therapy for skin burns. The question addressed here is to determine whether the culture process may induce cellular, molecular, or genetic alterations that might increase the risk of cellular transformation. Keratinocytes from four different human donors were investigated for molecular and cellular parameters indicative of transformation status, including (i) karyotype, (ii) telomere length, (iii) proliferation rate, (iv) epithelial-mesenchymal transition, (v) anchorage-independent growth potential, and (vi) tumorigenicity in nude mice. Results show that, despite increased cell survival in one keratinocyte strain, none of the cultures displayed characteristics of cell transformations, implying that the culture protocol does not generate artefacts leading to the selection of transformed cells. We conclude that the current protocol does not result in an increased risk of tumorigenicity of transplanted cells.

## 1. Introduction

In 1975, serial subculture of human keratinocytes was first described in [1]. Clinical application of this discovery was made possible after the preparation of these cells into epithelial sheets. Since then, cultured autologous and allogenic epithelia have been produced for treatment of extensive third-degree burns with good results [2]. Implementation of strict safety measures for banking allogenic keratinocytes has allowed minimizing the risks of infections and contaminations. However, there is still controversy as to whether the process of isolating and culturing keratinocytes prior to transplantation may somehow induce genetic modifications or enhance cell stem properties, potentially generating an increased risk of tumorigenesis which may be developed after transplantation. This question has been particularly difficult to address due to the lack of consensus on the cellular, molecular, or genetic parameters for assessing tumorigenicity in cultured keratinocytes. Recent developments on the knowledge of the sequence of molecular events required for transformation of primary epithelial cells *in vitro* have allowed us to select a set of defined parameters to allow such an assessment in a series of four independent cultures of primary keratinocytes.

The transformation of primary human cells is a multistep process that requires sequential alterations of several well-described pathways. According to Weinberg and collaborators [3–6], the transformation of primary cultures of epidermal cells requires the targeting of at least three mechanisms: combined inactivation of p53 and Rb pathways, constitutive activation of telomerase, and oncogenic activation of cell proliferation. These events have been rarely observed in primary human epithelial cell cultures. Primary keratinocytes preserve their capacity to enter replicating senescence associated with growth arrest and do not reactivate telomerase activity [7, 8]. On the other hand, inactivation of p53 and Rb pathways or spontaneous activation of Ras pathway have seldom been observed in such primary cultures, leading to the concept that human skin keratinocytes are resistant to transformation *in vitro*. One of the rare documented examples of spontaneous immortalization *in vitro* is the HaCaT cell line, which is derived from primary culture of adult skin. This cell line which has acquired mutations in *TP53*, is obviously immortal, has a transformed phenotype *in vitro* (clonogenic on plastic and in agar) but remains nontumorigenic *in vivo*. HaCaT cells, similar to normal keratinocytes, reform an orderly structured and differentiated epidermal tissue when transplanted onto nude mice [9]. It should be noted, however, that in primary cell culture, spontaneous immortalization *in vitro* is unlikely to occur during the very short time of culture (maximum 4 passages). In the case of HaCaT [9], immortalization required long-term *in vitro* culture conditions and is associated with a lag phase. During this phase, cells stop proliferating, enlarge, and remain growth arrested for a relatively long period (3–6 weeks). Thereafter, islands of small proliferating cells may appear and continue to grow without further interruption. Cellular transformation is thus preceded by an easily detectable phase of senescence associated with decreased growth rate. Then, in the case of transformation, epithelial cells undergo morphological changes and increase their growth rate [10] to form defined colonies. 

In the present study, we aimed at determining whether molecular, genetic, or cellular changes may occur during the short phase of primary culture of keratinocytes *in vitro*, which may not lead to a detectable culture phenotype but nevertheless affect the long-term tumorigenic potential of the cells. We have therefore used a range of approaches to check whether events related to the sequence of experimental transformation of human primary epidermal cells *in vitro* could spontaneously occur within cultures of primary keratinocytes processed according to protocols for allogenic transplantation.

## 2. Materials and Methods

### 2.1. Tissue Procurement

Normal human skin samples were obtained from the abdomen of four adult donors at the Edouard Herriot Hospital (Lyon, France) after informed consent of the patient. Donors were negative for HIV and hepatitis serology.

### 2.2. Cell Isolation

For epithelial cells extraction, the epidermis and underlying dermis compartments were separated by enzymatic digestion with thermolysin (overnight at 4°C). Epidermal cells were then isolated using a trypsin treatment at 37°C. Four cell strains were extracted: KAL0501 was the first strain out of 200 presenting morphological changes, KAL1152, KAL5045, and KAL0601 (one for each donor).

### 2.3. Cell Culture

Cells extracted from the epidermis were amplified on human fibroblast feeder layers until maximum passage at 10 000 cells/cm^2^. Keratinocytes were grown in Green medium [11] consisting of a 3 : 1 mixture of DMEM and Ham's F12, respectively, supplemented with 10% FCS, 10 ng/mL epidermal growth factor (EGF), 0.12 IU/mL insulin, 0.4 lg/mL hydrocortisone, 5 lg/mL triiodo-L-thyronine, 24.3 lg/mL adenine, and antibiotics.

### 2.4. Population Doubling (PD) and Growth Rate (GR)

Cells were seeded in three flasks, were resuspended after about 14 days by trypsin treatment, and were counted flask by flask:

(1) $$\begin{array}{cc} \text{PD} & =\frac{\ln\;\left(\text{cells number}/\text{CFU}\right)}{\ln\;2}\\ \text{GR} & =\frac{\text{PD}}{\text{Culture day number}} \end{array}$$

### 2.5. Telomere Length Assay

To determine telomere length, each strain was trypsinized at early (P1) and high passages (P5 or P17), and cell pellets were frozen at −80°C. Genomic DNA was extracted with QIAamp DNA Mini Kit and quantified by spectrophotometer. Telomere length was determined using Telo TAGGG Telomere Length Assay. Briefly, DNA was digested by HinfI and RsaI enzymes, DNA fragments were separated on agarose gel, transferred and hybridized with a telomere-specific digoxigenin- (DIG-) labeled hybridization probe. After chemiluminescence detection, results were compared with DIG molecular weight markers and two control DNAs (short and long telomeres).

### 2.6. Chromosomal Analysis

For this experiment, cells were cultured without feeder layer, in keratinocytes serum-free medium supplemented with 25 mg of bovine pituitary extract and 2.5 *μ*g of EGF and antibiotics. Standard cytogenetic techniques using GTG and RHG-banding were performed for chromosome examination. The analyses were performed on 15 mitosis per strain.

### 2.7. Tp53 Sequencing

For mutation detection, DNA was extracted from the four cell strains at passage 5.TP53 mutation analysis of exons 5–8 was performed by PCR-based direct sequencing. The following forward (F) and reverse (R) primers were used:

 5F(5′TGTTCACTTGTGCCCTGACT3′) 5R(5′CAGCCCTGTCGTCTCTCCAG3′)
 6F(5′GCCTCTGATTCCTCACTGAT  3′) 6R(5′TTAACCCCTCCTCCCAGAGA  3′)
 7F(CTTGCCACAGGTCTCCCCAA3′) 7R(5′AGGGGTCAGCGGCAAGCAGA  3′)
 8F(5′TTCCTTACTGCCTCTTGCTT  3′) 8R(5′AGGCATAACTGCACCCTTGG  3′)

PCR conditions were as follows: for exons 5, 6, and 8, denaturation at 94°C for 2 minutes followed by annealing for 45 seconds at temperatures from 63°C to 60°C, decreasing 0.5°C every 3 cycles and extension at 72°C for 1 minute. For exon 7, the selected condition was at a denaturation temperature of 95°C for 15 minutes followed by 94°C for 30 seconds, annealing at 60°C for 30 seconds and extension at 72°C for 1 minute. Aliquots of PCR products were examined by electrophoresis on 2% agarose gel containing ethidium bromide. PCR products were treated with 2 *μ*L ExoSAP-IT at 37°C for 15 minutes followed by inactivation at 80°C for 15 minutes and directly sequenced using Applied Big Dye Terminator v1.1 cycle sequencing method on ABI Prism 3100 Genetic Analyzer. The sequences obtained were compared with the reference sequence, X54156, from Genbank (http://p53.iarc.fr/TP53sequenceX54156.html). *KRAS *mutations at codon 12 were analyzed by direct sequencing of exon 1 as described elsewhere [12].

### 2.8. Detection of Epithelial-Mesenchymal Transition by Western Blotting

Epithelial-mesenchymal transition (EMT) was analyzed in keratinocyte cultures by measuring E-cadherin and vimentin expression by Western blotting. Cultured keratinocytes at early (P1) and high passages (P5) were trypsinized and collected by centrifugation. The resulting cell pellets were frozen at −80°C until analyses. Cells were lysed in lysis buffer [50 mM Tris HCl, pH 7.4, 250 mM NaCl, 0.1% SDS, 0.5% Nonidet P-40 (NP40) containing protease inhibitors (2 *μ*g/mL aprotinin, 500 *μ*M phenylmethylsulfonyl fluoride, 0.5 *μ*g/mL leupeptin, 1 pg/mL pepstatin and 2 mM dithiothreitol)] and placed on ice for 30 minutes. Extracts were subsequently centrifuged at 13 000 rpm for 15 minutes at 4°C. Supernatants were collected. Protein concentration was determined using Bradford reagent. Thirty*μ*g of protein extracts were separated on 7.5% SDS-PAGE and transferred to Polyvinylidine difluoride membranes by electroblotting. Membranes were blocked with 5% dry milk in PBS-NP40 (0.05%) for 1 hour at room temperature and incubated overnight with primary antibody diluted in 1% PBS -NP40 0.001% containing 1% dry milk. The following antibodies were used: anti-E-cadherin clone 36 (Becton-Dickinson) at a concentration of 0.25 *μ*g/ml, and antivimentin clone V9 (Dako) at a dilution of 1/500. The membranes were washed and incubated with peroxidase—conjugated secondary antimouse IgG antibody (Dako) at a dilution of 1/1000. The signals were detected by enhanced ECL kit (Amersham).

### 2.9. Detection of Epithelial-Mesenchymal Transition by Immunofluorescence

Cells were seeded and cultured on coverslips in 12-well plates. At subconfluence, cells were fixed in 4% formaldehyde in PBS at room temperature for 15 minutes, rinsed by several changes of PBS. Cells were permeabilized in Triton X100 1% in PBS for 5 minutes and rinsed 3 times 5 minutes in PBS. Cells were blocked 30 minutes in FBS 10% at room temperature and subsequently exposed to the primary antibodies raised against vimentin (1/50), E-cadherin (1/250) for 2 hours and then to secondary goat antimouse (1/200) conjugated FITC for 1 hour. The fluorescent samples were examined with a Zeiss laser scanning confocal microscope LSM 510 on Axiovert 200 M. The LSM 4.0 software was used for image acquisition and analysis.

### 2.10. Soft Agar Assay

To determine the anchorage-independent growth potential, the colony formation was measured in soft agar. Cells of each strain were seeded into 75 cm² culture dishes and grown to 60%–70% confluence. 0.75% and 0.45% agar media were prepared by mixing 1.5% or 0.9% agar, respectively, and DMEM-HAM's F12 2X with 20% FBS at a proportion 1 : 1. For the underlayer, 1.5 mL of 0.75% agar medium was added to 60 mm wells. 293T cells were used as positive control. They were generated by transformation of cultures of normal human embryonic kidney cells with sheared adenovirus and transfected by SV40 Large T-antigen. These cells are able to grow in soft agar and induced tumors in nude mice [13]. Cells at P5 for KAL 0601, 5045, 1152 and at P21 for KAL 0501 were harvested by incubation in trypsin-EDTA, centrifuged at 1000 rpm, and 5000 cells were suspended in 1.5 mL of 0.45% agar medium. Cell suspensions were then plated on the underlayer of 0.75% agar. Agar plates were incubated at 37°C for 3 weeks. Cell colonies were scored using an inverted microscope. As a rule, cell clusters measuring more than 80 *μ*m in greatest diameter were considered a colony. Each soft agar assay was performed in triplicate.

### 2.11. Tumorigenicity Assay in Nude Mice

Female athymic mice were selected aged 6 weeks (nu/nu genotype), from Charles River (Lyon, France). After 3 weeks of quarantine and acclimation for at least 3 days, mice were placed in an animal facility compliant with regulation. The HPKIA cell line at passage 345, a transformed cell line, was used as a positive control, and HPKIA at passage 28, nontumorigenic, were used as a negative control [14]. The keratinocytes and the HPKIA tumor cells as reference strains were harvested, washed with serum free medium and counted. The inoculum of each donor was subcutaneously injected in 3 nude mice near the scapula. Each animal received 5 × 10^6^ cells suspended in 0.2 mL of PBS. The different cell strains were injected into separate groups. All the animals, including the reference group, were observed and examined once a week for the formation of nodules at the sites of injection. The period of observation was three months, and at the end all animals including the reference group were killed and autopsied. The injection site area, kidney, lung, liver, and lymph node were embedded in paraffin blocks and were subjected to histopathological examination.

## 3. Results

### 3.1. Clonogenicity, Growth Rate and Population Doubling

**Figure 1:**
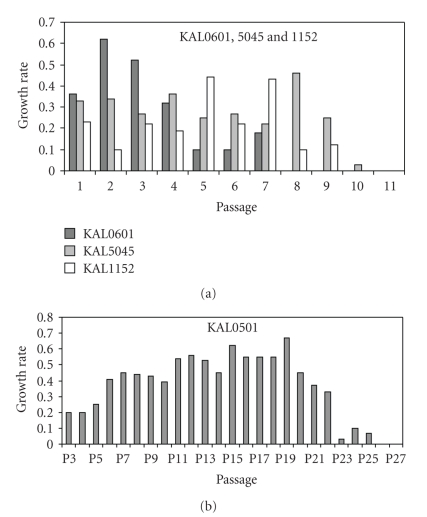
Growth rates of keratinocytes in function of passages. (a): growth rate of KAL 0501 from P1.

Cells KAL0601, 5045, and 1152 displayed normal growth patterns, with homogenous clones composed of small cells tightly attached to each other. This pattern did not change with culture time or passaging. As expected, growth rate (GR) and population doubling (PD) of these epidermal cells decreased with passaging. Nevertheless, in P5, cells presented no sign of crisis, and they could grow approximately until the passage P10 before displaying a senescent phenotype (Figure 1(b)). 

**Figure 2:**
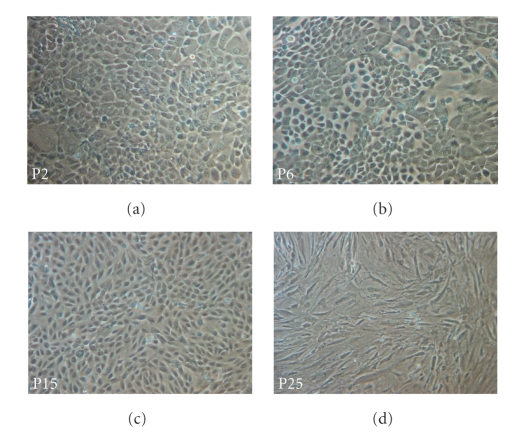
Pictures of KAL0501 in culture at different passages (x100). Primary keratinocytes KAL0501 are culture in green medium on human feeder layer. Their morphology has changed during the culture from passage P1 to P25.

Cells KAL0501 displayed a particular growth pattern. Between passages P0 and P3, the morphology of these keratinocytes remained homogeneous with a characteristic pavement shape and strong ties between cells (Figure 2(a)). From P4 to P5, cells increased in size and assumed a heterogeneous aspect. However, cells appeared to retain the capacity to attach to each other. At P6 (Figure 2(b)), among normal clones, some cells appeared to lose this attachment capacity and, between P6 and P10, these cells of abnormal morphology gradually invaded the culture. From P10 to P18 (Figure 2(c)), cells either took a spindle-shaped aspect when they had enough space to stretch out, or they appear to revert to a pavement-like shape when short of space. Furthermore, cells did not have a clonal growth in the latest passages, but appeared to grow independently of each other (Figure 2(d)), After settling in culture at P0, cells initially showed a progressive decrease in their GR into P5 and then presented signs of crisis. From P5 to P15 the cells bypassed the crisis with an increase of their PD and GR. However, after P15, their GR decreased slowly with passaging until death at P25 (Figure 1(a)). 

### 3.2. Telomere Length Assay

Telomere length was determined for each strain at low (P1) and high passages (P5) for KAL5045, 1152, 0601 and P17 for KAL050 (Table 1). For all strains and whatever the passage analyzed, telomere length was as long as the high telomere length control of 10.2 Kb, in agreement with the fact that cells were in a proliferating stage. For KAL 0501 at P17, however, telomere length has decreased, consistent with the observation that these cells had initiated a senescence process and were not immortalized.

### 3.3. Chromosomal Analysis

**Figure 3:**
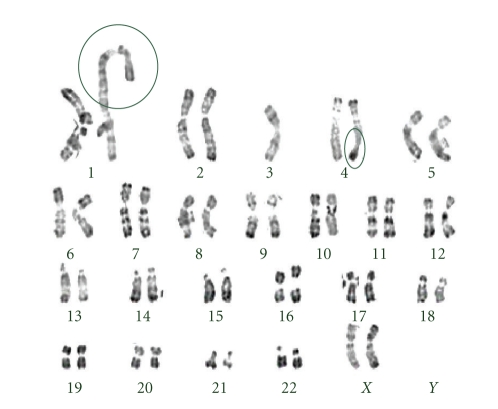
Karyotype of KAL0501 at passage P20. At this passage, two abnormalities are observed: an unbalanced translocation 4q32 and duplication 1q, or a translocation 1q with another chromosome.

Continuous serial passaging of any cell type can eventually lead to chromosomal rearrangements, sometimes resulting in a transformed phenotype. To assess chromosomal stability, the karyotypes of KAL0601, 5045, and 1152 were evaluated at P3 and P4. They showed normal karyotypes. As for KAL0501, no chromosome abnormalities were detected at P1 or P10. However, some structural abnormalities were detected at P20 (Figure 3). These abnormalities include (i) an unbalanced translocation involving 4q32, and (ii) a duplication at 1q, or a translocation 1q with another chromosome. In the case of chromosome 4q32, there was a deletion of the terminal q region, with partial monosomy of this chromosome associated with a partial trisomy of another chromosome. At P15, out of 15 mitoses analyzed, two presented the abnormality of the long arm of the chromosome 4, but none of them presented the chromosome 1 abnormality. Thus, we suggest that the structural changes in 4q32 may have occurred between P10 and P15, whereas the changes in 1q may have taken place later, between P15 and P20.

### 3.4. Somatic Alterations in Cancer-Related Pathways

Mutations in the tumor suppressor gene TP53 are frequent in human cancers. The TP53 database (www.iarc.fr/P53/) compiles all mutations (somatic and inherited), as well as polymorphisms, that have been reported in the published literature since 1989. Exons 5 to 9 encode the DNA-binding domain containing over 90% of all mutations in human cancers and only a few SNPs (with a low frequency) occuring in this region [15, 16]. No *TP53* mutation or polymorphism were detected in exons 5 to 9 in any of the four cell strains, including KAL0501 at P5 and P20 (data not shown). Mutations in codon 12 of *KRAS* are one of the most common mechanisms of activation of ras-related pathways in human cancers. Sequencing of KRAS exon 1 (encompassing codon 12) did not reveal any mutation (data not shown). Similarly, Western Blotting for effectors of cell-cycle regulation such as p16INK4a or Rb showed normal expression patterns in all cell strains (data not shown). Therefore, none of the cell strains presented with somatic alterations are characteristic of the sequence of transformation of primary human epithelial cells *in vitro.*

### 3.5. E-Cadherin and Vimentin Expression

In the course of tumor progression, epithelial cells undergo epithelio-mesenchymal transition (EMT) during which cells acquire a fibroblast-like phenotype and the ability for directed migration and become dissociated from each other. These events are the basis for invasion and metastasis of malignant cells. EMT may occur at the very first steps of cell transformation. To evaluate the occurrence of EMT, expression of E-cadherin, a specific marker of epithelial cells, and expression of vimentin, a specific marker of mesenchymal cells, were investigated by western blotting on keratinocytes cultures at passages 1 and 5 for KAL 0601, 5045 and 1152. The results show that these cells express normal levels of E-cadherin but lack vimentin expression, at both passages tested (data not shown).

**Figure 4:**
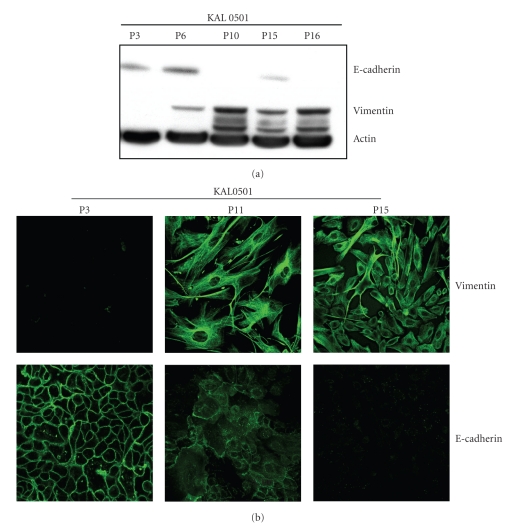
Expression of vimentin and E-cadherin in KAL0501 at different passages in western blot and immunofluorescence. (a): E-cadherin expression level of KAL 0501 decrease from P3 to P16 on contrary to vimentin expression which increase. (b): picture (confocal microscope x25) of KAL0501 at P3, P11, and P15 cultured on monolayer. E-cadherin disappears progressively with the passage and vimentin appear at P11.

With respect to KAL0501, Figure 4(a) shows the results obtained at passages P3, P6, P10, P15, and P16. The expression of E-cadherin was normal from P3 to P6, was strongly decreased at P10, and disappeared at P16. However, it was still detectable until P15. As described above, until P6 KAL0501, it tended to adopt an epithelial-like morphology, which may explain the persistence of E-cadherin expression. In contrast, at P16, cells adopted a spindle-shape, with intense vimentin expression. These results on KAL0501 were confirmed by immunofluorescence (Figure 4(b)). showing increased expression of vimentin from P3 to P11 and P15 and a progressive decrease of E-cadherin expression. In contrast to Western Blotting, however, E-cadherin was not detected at P15 suggesting that its expression was extremely low.

### 3.6. Soft Agar Assay

**Figure 5:**
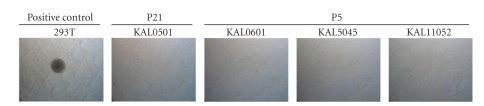
Picture of soft agar assay (Microscope x10). The 4 cell strains are not able to grow in soft agar after 21 days of culture on contrary to the positive control, the transformed human embryonic kidney 293T cells.

The test on soft agar allows the study of cells capacity to multiply without adhering to a solid support, a characteristic of transformed cells. This test was performed on a transformed cell line (positive control, 293T cells), on KAL0501 at passages P21, and on KAL 0601, 5045, and 1152 at passage P5. Results are presented in Figure 5. After 21 days of culture, it was observed that the positive control grew in clones in agar and gave rise to 76 clones of at least 80 *μ*m diameter per well after 5 weeks of culture. With respect to KAL0501, no cells were able to adhere nor to form clones on agar at P21. Neither could keratinocytes KAL0601, 5045, and 1152 grow on soft agar at P5.

### 3.7. Tumorigenicity and Experimental Metastasis Assay

Cultured keratinocytes KAL0501 at P22, KAL5045 at P6, KAL1152 at P5, and KAL0601 at P5 were injected in nude mice, and tumor formation was monitored for at least three months. As control, we used the HPV16-immortalized keratinocyte cell line HPKIA which has acquired the ability to form squamous cell carcinomas in nude mice after gamma irradiation and long-term culturing *in vitro*. This cell line expresses the oncoproteins E6 and E7 of human papillomavirus type 16 (HPV16) [14]. Although becoming immortal *in vitro* after a limited number of passages (P28), HPKIA do not become tumorigenic *in vivo* until much later passages (P345), allowing to use early or late passages of these cells as negative or positive controls of tumorigenicity, respectively. Three months after injection, all mice receiving positive tumor control, developed rachitis and displayed tumors at the injection points. In contrast, the 3 mice which received the negative control did not reveal any tumors at the injection site and were in good health. Mice that received 5 million of any of the four strains of keratinocytes, including KAL0501 at P22, were in good health without rachitis and did not present any tumor or nodule by palpation throughout the observation period. Similarly, no tumor was found at autopsy. After histopathological examination, only mice injected with HPKIA at P345 displayed well-differentiated keratinized squamous cell carcinoma followed by lung metastasis. All other mice did not show any microscopical evidence of tumors* in situ *or of metastasis.

## 4. Discussion

The spontaneous transformation of human skin keratinocytes *in vitro* is a rare event. Since 1981, epidermal cell sheets produced *in vitro* have been used for grafting in the treatment of severely burned patients, and no cases of cancer or occurrence of hyperproliferative lesions in relation with the graft have been reported in the literature. Only one case of squamous cell carcinoma of the skin occurring within an area of grafted skin has been reported [17]. In this case, however, the graft consisted of full-thickness skin taken from the right postauricular area to resurface a lesion generated by the incomplete excision of a basal cell carcinoma. In our laboratory, primary keratinocytes have been cultured since 1988 with cultures developed from more than 200 different donors. The only example of a culture that spontaneously developed a modified pattern *in vitro* is the cell strain KAL0501 which is reported herein. This easily recognizable abnormal cell morphology has led to its elimination from clinical applications and all present investigations in order to identify molecular markers that may help to detect the earliest changes leading to transformation in primary cultures of skin keratinocytes. 

When compared to 3 other strains of keratinocytes representative of the types of cultures routinely generated in the laboratory, KAL0501 presented a specific phenotype. Phenotypic changes were sporadically observed from P2. The cell contours became refringent, unlike normal cells, with dark contours when cultured on a feeder layer. Until P5, however, cell growth decreased as for normal cultures. However, in contrast to normal cultures, this decrease was followed by resumption of growth with loss of differentiation and appearance of vimentin expression. At this passage P5, intercellular junctions became inefficient and these cells became unable to form cohesive epidermal sheets in culture (data not shown). Despite detection of a chromosomal translocation at 4q32, KAL0501 did not appear to develop a capacity for long-term growth *in vitro*, did not form colonies in soft agar, and did not form tumors after injection in nude mice. We therefore conclude that despite EMT, phenotypic, and chromosomal changes, KAL0501 did not undergo immortalization *in vitro* and did not acquire tumorigenic properties. Therefore, despite partial lack of differentiation *in vitro*, KAL0501 does not exhibit the properties of a transformed cell line. 

In cell culture, it is extremely unlikely that during the limited time of culture (4 passages maximum) keratinocytes accumulate any mutations that could lead to the settlement of a neoplastic phenotype. To confirm this hypothesis, we have analyzed TP53 (exons 5–8) and KRAS (exon 1) by sequencing two of the genes that are most frequently mutated in human epithelial tumors. Although our analyses do not encompass the whole gene sequence, it covers all cancer-related mutational hotspots as well as about 90% of the missense mutations reported for these genes in human cancer. We did not find evidence for such mutations, including in the KAL0501 strain. Western blotting of proteins involved in the Retinoblastoma pathway, such as RB itself and p16INK4a, did not reveal any abnormality at the protein level. Telomere length in cells at P5 was also analyzed. Reduction of telomere length is one of the hallmarks of progression of primary cells in culture towards replicative senescence. All cell strains at P5 presented with long telomeres, consistent with the notion that at P5 they are still at the proliferative stage. KAL0501 cells were not different from other cells in this respect, and their telomere length decreased at high passage, consistent with the fact that these cells initiated a senescence process and were not immortalized. To ensure that transformation may not occur as the result of a contamination of these cells by oncogenic human papillomaviruses, the viral DNA for serotypes 16, 18, 26, 31, 33, 35, 39, 45, 51, 52, 53, 56, 58, 59, 66, 68, 70, 73, and 82 were investigated by multiplex PCR method combined with DNA microarray primer extension [18]. None of the strains, including KAL0501 showed evidence of papillomavirus infection at passages 1, 3, 5, and 10 (data not shown). Overall, we conclude that none of the four strains presented obvious characteristics of early steps of transformation. 

Despite the absence of immortalization *in vitro* and of tumorigenicity *in vivo*, the KAL0501 strain showed remarkable changes in its differentiation pattern, with a capacity to undergo at least some of the phenotypic changes associated with EMT. The molecular bases for this property are unknown and may deserve further investigations. Interestingly, the karyotype of KAL0501 reveals a translocation inducing a loss of the terminal part of chromosome 4q, which may have occurred at around passage 10–15 in this particular strain. This area is a common area of loss in many cancers and developmental diseases. In particular, it has recently been proposed as a possible area for genes involved in SHFM (Split Hand and Foot Malformation Syndrome), a developmental defect characterized by abnormal epidermal to mesenchyme interactions during morphogenesis [19]. It is tempting to speculate that this region may contain some gene(s) or relevance for the regulation of EMT. 

With clinical applications in mind, it is essential to make sure that the protocols used for producing epidermal sheets for transplantations are safe not only from the point of view of the risk of contamination and of immune response, but also in terms of long-term cell stability. Our results concur with the notion that human keratinocytes are extremely stable *in vitro* and may not experience transforming mutations during the short time of *ex vivo* expansion (4 passages maximum). However, as demonstrated by the case of the KAL0501 strain, significant morphological changes may occur, during which some cultures may adopt phenotypes demonstrating altered differentiation and enhanced growth properties. Such changes are easy to detect as their first effects are to modify the normal clonal growth pattern, and prevent the formation of tight epithelial sheets. They may therefore be detected and eliminated before grafting.

## 5. Conclusion

Transformation of human epithelial cells *in vitro* is a rare event and difficult, in part due to the tight control over genetic and genomic stability and to the limited replicative lifespan of primary human cells. In the present study, we demonstrate that different strains of primary human keratinocytes do not show any evidence of transformation and tumorigenicity even if one of them, KAL0501, develops an altered growth pattern with features that resemble epidermal to mesenchyme transition. Of note, this strain is the only one presenting such a phenotype among a series of over 200 independent cultures developed in our laboratory since 1988. Therefore, we conclude that the protocols used for generating keratinocyte sheets for transplantations are reasonably safe with respect to their long-term stability and lack of tumorigenicity. Nevertheless, alterations in cell shape, adhesion and growth patterns may occur in rare instances, calling for special attention before using cultures for clinical use.

## Figures and Tables

**Table 1:** Telomere length of keratinocytes.

Cell strain	Culture passage	Telomere length (Kpb)
KAL 0501	1	10,9
KAL 0501	17	7,6
KAL 1152	1	10.6
KAL 1152	5	10.3
KAL 5045	1	10.6
KAL 5045	5	9.8
KAL 0601	1	9.8
KAL 0601	5	8.7

Control low telomere length		3.9
Control high telomere length		10.6
